# Action Mechanism, Research Progress and Development Trend of High-Temperature Steam Flooding and Profile Control/Flooding Systems

**DOI:** 10.3390/gels12070586

**Published:** 2026-07-02

**Authors:** Yigang Liu, Jianhua Bai, Xiaodong Han, Qiuxia Wang, Hongwen Zhang, Hongyu Wang, Jinxiang Liu, Yifei Gao, Xianpei Yin, Zilong Liu

**Affiliations:** 1 CNOOC Key Laboratory of Offshore Heavy Oil Thermal Recovery, Tianjin 300452, China; liuyigangdbsy@163.com (Y.L.); baijianhuadbsy@163.com (J.B.); hanxiaodongdbsy@163.com (X.H.); wangqiuxiadbsy@163.com (Q.W.); zhanghongwendbsy@163.com (H.Z.); wanghongyudbsy@163.com (H.W.); 2 Key Laboratory of Enhanced Oil and Gas Recovery of Ministry of Education, Northeast Petroleum University, Daqing 163318, China; gyf20000224@163.com (Y.G.); yinxianpei620@163.com (X.Y.); zliul12@163.com (Z.L.)

**Keywords:** gels, high-temperature steam flooding, profile control and flooding, thermally responsive materials, conformance control, enhanced oil recovery

## Abstract

Offshore high-temperature steam flooding suffers severe steam channeling, uneven steam intake and low thermal efficiency, while conventional profile control agents fail to adapt to coupled harsh environments of 200–350 °C high temperature, ultra-high salinity and continuous steam shear. Existing reviews mainly focus on onshore thermal reservoirs or single foam/gel materials, lacking a targeted, gel-oriented systematic review matching unique offshore platform constraints. Guided by the integrated framework of “flow control–diversion–enhanced sweep efficiency”, this work establishes a six-dimensional quantitative screening standard and unified performance comparison database to systematically review foam, gel, particle, thermo-responsive and composite profile control systems. Differing from petroleum engineering-oriented summaries, this paper subdivides high-temperature gels into six categories from a polymer material perspective, elaborating their crosslinking mechanisms, thermal rheology and cyclic steam degradation rules; the inherent advantages, limitations and offshore applicable boundaries of each medium are quantitatively compared, with special emphasis on the unique “deep migration followed by in situ thermal activation” mechanism of thermo-responsive materials. Composite systems relieve single-material defects via multi-mechanism synergy yet face complicated on-site deployment barriers. Three core bottlenecks restricting field application are identified: the irreconcilable trade-off between deep propagation and stable plugging, large deviation between static aging results and dynamic anti-scouring performance, and exclusive engineering limitations of offshore platforms. A dedicated standardized dynamic laboratory evaluation scheme for cyclic steam flooding is proposed to narrow lab-field performance gaps. Future research priorities include salt-resistant thermally responsive composite gel modification, low-cost multi-component compound formula optimization, unified dynamic evaluation criteria and staged material matching guidelines to realize balanced performance of high-temperature tolerance, deep delivery and offshore operability.

## 1. Introduction

Heavy oil resources are core alternative reserves for offshore oil and gas development in China [1]. High crude viscosity and severe reservoir heterogeneity render conventional exploitation methods inefficient. Steam flooding, a mature follow-up thermal EOR technology after cyclic steam stimulation [2], relies on uniform steam propagation to achieve economic heavy oil recovery, yet its field effect is severely restricted by steam channeling, uneven steam absorption profiles and low heat utilization efficiency [3,4,5].

Reservoir permeability differences create permanent high-permeability dominant flow channels. Coupled with steam gravity override and gravitational differentiation, steam preferentially migrates upward along high-permeability zones, leaving medium–low permeability intervals insufficiently heated and swept, which widens inter-well development gaps and raises operation costs.

Offshore platforms impose additional unique constraints absent from onshore oilfields: limited deck space, compact injection preparation equipment, extremely high single-well operation costs, and tight operation windows. Meanwhile, offshore formation water is characterized by high total dissolved solids (TDS) and complex ion compositions, which accelerate polymer chain hydrolysis, break crosslinking bonds, and induce structural collapse of conventional polymer plugging agents. Traditional onshore profile control and water shutoff systems cannot adapt to the coupled harsh environment of 200–350 °C continuous steam scouring, high salinity, and long-distance inter-well fluid migration. Developing dedicated offshore profile control and flooding systems is therefore critical to improving thermal recovery performance.

**Figure 1:**
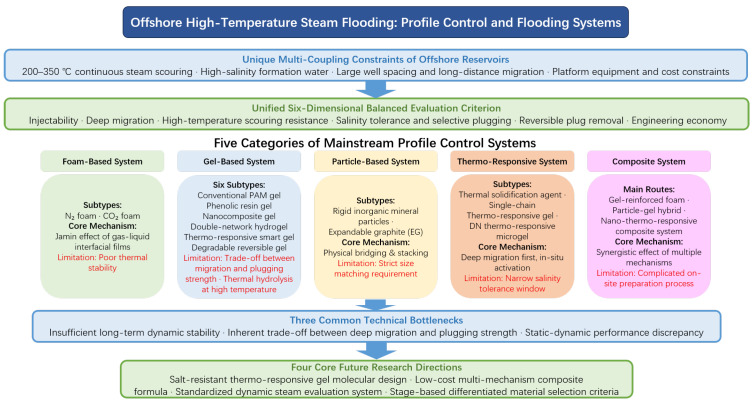
Analytical framework and classification system of profile control and flooding systems for offshore high-temperature steam flooding.

Aiming at the above multi-coupling constraints, this review systematically sorts out five categories of mainstream profile control systems, establishes a unified six-dimensional balanced evaluation criterion, summarizes three common technical bottlenecks, and proposes four core future research directions. The overall logical framework of this work is shown in Figure 1.

### 1.1. Research Gaps in Existing Reviews

Previous literature has separately summarized gel plugging materials, steam foam auxiliary flooding, thermal EOR conformance control and onshore heavy oil profile control technologies, but five prominent research blind spots remain, which necessitate a targeted review focusing exclusively on offshore high-temperature steam flooding profile control systems:

1. Existing gel reviews target onshore thermal reservoirs and ignore offshore coupling constraints. Extensive thermal EOR reviews have summarized material optimization and field application experience for onshore conventional reservoirs [6]. However, most gel-centered reviews only discuss polymer/phenolic gels under conventional land thermal recovery, focusing merely on static thermal stability. They overlook dynamic high-speed steam shear, cyclic thermal aging failure, offshore narrow injection windows and platform equipment limits. Few independently classify nanocomposite, double-network and thermosensitive hydrogel branches nor systematically elaborate their high-temperature crosslinking kinetics and thermorheological properties from a polymer material perspective, failing to match the material research orientation of the *Gels* journal.
2. Foam and thermal EOR reviews lack a unified cross-system quantitative comparison framework. Studies on steam foam or general thermal conformance control only analyze foam, gel or particle media in isolation. No unified evaluation system covering temperature resistance, plugging efficiency, oil increment, injectability and offshore field adaptability has been built, nor are the applicable reservoir stages and boundary constraints of each medium clearly defined.
3. Onshore heavy oil profile control summaries cannot be directly applied to offshore scenarios. Current reviews mainly summarize profile control technologies for onshore cyclic steam stimulation and onshore steam flooding, ignoring offshore distinctive features: large well spacing horizontal well groups, long-distance inter-well fluid transport, strict injection fluid viscosity/particle size limits and high construction costs. Field experience from onshore pilot tests cannot be copied offshore, while no literature systematically sorts the matching rules between profile control formulas and offshore engineering boundaries.
4. Insufficient systematic discussion on static–dynamic performance deviation and standardized offshore laboratory evaluation. Prior works briefly mention that many materials perform excellently in static high-temperature aging tests but degrade rapidly under dynamic steam flow, without independent in-depth analysis of dynamic core-flood protocols, cyclic steam aging degradation, thermo-mechanical coupled failure and offshore-oriented reservoir numerical simulation schemes. No unified guidance is provided to narrow the performance gap between indoor evaluation and field practice.
5. Separation of material chemistry mechanisms and offshore engineering application in past literature. Earlier studies fall into two isolated categories: material papers only elaborate synthesis and crosslinking mechanisms, while engineering reviews merely summarize field construction effects. No integrated analysis logic of “material mechanism–laboratory performance–offshore applicability” guided by the unified “flow control–diversion–enhanced sweep efficiency” theory has been proposed.

### 1.2. Unique Coupled Restrictive Boundaries of Offshore High-Temperature Steam Flooding

Three core differences distinguish offshore thermal recovery from onshore steam flooding and raise higher comprehensive requirements for profile control agents:

1. Multi-coupled harsh reservoir environment: Long-term continuous scouring by 200–350 °C steam, high-salinity formation water, severe interlayer/lateral heterogeneity and frequent inter-well steam breakthrough.
2. Special offshore platform engineering limits: Compact equipment layout, strict restrictions on injection fluid viscosity and particle size, high single-round operation cost and short valid construction window [7];
3. Exclusive offshore development mode: Large-spacing horizontal well groups, long-distance inter-well fluid migration, multi-cycle repeated steam injection and persistent heat loss between wells.

General reviews focusing on onshore thermal recovery or single foam/gel materials cannot fully cover the above multi-dimensional constraints, which highlights the necessity of this specialized integrated review.

### 1.3. Innovative Contributions of This Review

Against the above research deficiencies, this work establishes the following three distinct novel perspectives that differentiate it from all existing related reviews, as quantitatively summarized in Table 1:

1. A unified quantitative multi-system comparison framework + targeted offshore material screening criteria. This paper constructs a complete statistical table covering foam, conventional polymer gel, phenolic-resin gel, nanocomposite/double-network/thermosensitive gel, inorganic particle and multi-component composite systems, with unified quantitative metrics of temperature tolerance, salinity resistance, plugging efficiency, incremental oil recovery, injectability, anti-scouring performance, strengths and offshore limitations. Combined with platform operation constraints, this review proposes systematic material selection standards absent from previous summaries.
2. In-depth material science analysis matching the positioning of *Gels*. Unlike petroleum engineering-oriented reviews that simplify gels as simple plugging agents, this paper subdivides high-temperature gel systems into six independent subtypes. It systematically analyzes high-temperature crosslinking pathways, thermally induced network degradation and rheological evolution under sustained steam thermal stress, focusing on responsive gel material characteristics that constitute the core scope of the journal *Gels*.
3. Independent special subsection for dynamic steam performance evaluation + clear offshore future research roadmap. This review sets an independent section to discuss static–dynamic performance mismatch, standardized dynamic core-flood testing procedures, cyclic steam aging degradation mechanisms and optimized laboratory evaluation schemes tailored for offshore conditions. Based on the two universal bottlenecks of existing systems (insufficient long-term dynamic stability, inherent contradiction between deep migration and effective plugging), four targeted offshore-oriented research directions are put forward to guide subsequent material modification and field matching studies.

Table 1 clearly demonstrates that all existing reviews show obvious one-sidedness in research scope and analytical depth. Most prior works only focus on single gel/foam materials or summarize onshore plugging technologies, lacking an integrated analysis system combining gel polymer material chemistry, cross-system quantitative horizontal comparison and offshore dynamic steam evaluation. This paper remedies the above multi-dimensional research blind spots through systematic induction and critical comparative discussion, forming a specialized analytical review oriented to offshore high-temperature steam flooding profile control materials.

Combined with the sorted research gaps and innovative positioning above, Section 2 systematically elaborates the functional demands, standardized design criteria, internal action mechanisms, performance advantages and offshore matching limits of five mainstream profile control media.

## 2. Requirements and Design Principles of Profile Control and Flooding Systems for Offshore High-Temperature Steam Flooding

### 2.1. Core Contradictions Restricting Offshore Plugging Agent Performance

The performance limitations of conventional profile control agents arise from three mutually coupled contradictions unique to offshore steam flooding, which have not been fully addressed in onshore thermal recovery reviews [3,5,17].

1. Reservoir intrinsic contradiction

Reservoir permeability heterogeneity creates inherent permeability differences between interlayers and lateral zones. Under long-term continuous high-temperature steam erosion, high-permeability zones gradually evolve into irreversible dominant steam channels, as verified by multiple reservoir simulation and field monitoring studies [4,18]. Combined with steam gravity override and gravitational differentiation, steam preferentially migrates upward along high-permeability intervals, leaving medium–low permeability oil layers insufficiently heated and swept [19]. Offshore large well spacing further amplifies the severity of inter-well steam channeling and widens development differences across well groups, which cannot be eliminated by simply increasing steam injection volume [17].

2. Material performance trade-off contradiction

All single-component foam, gel and particle systems face an unavoidable inherent trade-off: low initial viscosity required for deep migration conflicts with the high structural strength needed for stable plugging [13,20]. Conventional static high-temperature aging tests generally overestimate material service life, as cyclic dynamic steam shear causes progressive structural degradation that static experiments cannot reproduce, creating a notable evaluation deviation between laboratory results and field performance [21,22].

3. Offshore engineering constraint contradiction

Compact platform layout limits the scale of injection and mixing equipment, making complex multi-stage preparation processes impractical [17]. High single-operation costs and short construction windows rule out the repeated profile control treatments commonly adopted on land. Meanwhile, offshore high-salinity formation water accelerates hydrolysis and crosslink network collapse of traditional polymer plugging agents, a factor seldom emphasized in onshore-oriented literature [5,8].

To resolve the above multi-dimensional conflicts, profile control systems should be developed under the unified guidance of the “flow control–diversion–enhanced sweep efficiency” framework. Balancing migration capacity, anti-scouring stability and on-site operability serves as the core evaluation criterion for cross-system comparison in subsequent chapters.

### 2.2. Functional Orientation and Six-Dimensional Balanced Evaluation Indicators

Unlike conventional water shutoff agents that pursue rigid permanent plugging, offshore steam flooding profile control agents target dynamic steam flow redistribution, a distinction rarely clarified in previous single-material reviews [8,12]. Their three coordinated core functions, widely validated by laboratory and field data [14,23,24], directly correspond to the “flow control–diversion–sweep enhancement” theory as follows:

(1) Reversibly increase flow resistance in high-permeability channels without completely blocking pore throats to avoid permanent reservoir damage;
(2) Force steam to divert into underutilized low-permeability layers, which is the core EOR contribution of profile control treatment verified by parallel core flooding experiments [20,24];
(3) Maintain stable regulation performance after multiple rounds of cyclic steam injection, rather than failing within a single injection cycle.

Drawing on established experimental standards [13,25,26], six mutually restrictive quantitative evaluation indicators are established for offshore material screening, forming a complete balanced screening system absent from fragmented single-factor studies.

1. Injectability: Low initial viscosity and uniform particle dispersion to avoid pipeline plugging and near-wellbore accumulation, adapting to limited offshore mixing equipment [17].
2. Deep migration capacity: Delayed gelation, particle deposition or thermal activation to achieve long-distance transport to deep inter-well dominant channels [13,20].
3. High-temperature anti-scouring stability: Resistance to long-term thermal degradation and repeated steam shear at 200–350 °C; dynamic anti-scouring performance takes priority over static aging results [15,22].
4. Salinity tolerance and selective plugging: Resistance to structural damage from high-salinity formation water; selective plugging of high-permeability zones while minimizing damage to productive low-permeability layers [14].
5. Reversible plug removal performance: Controllable degradable structures or reversible crosslinking networks to facilitate later reservoir adjustment without permanent formation damage [8].
6. Engineering economy and operability: Low-cost, easily accessible raw materials and simple mixing workflows matching the short construction windows of offshore platforms [17].

Optimizing any single indicator often comes at the cost of others: higher crosslink density improves thermal stability but impairs injectability and migration capacity; ultra-fine particles extend migration distance yet lose bridging plugging efficiency. This mutual restriction explains why single foam/gel/particle materials cannot simultaneously satisfy all offshore operating demands.

### 2.3. Balanced Design Principles for Offshore Profile Control Systems

The six design principles are mutually restrictive and balanced, serving as the unified benchmark for comparing all profile control media in Chapter 3. Most existing laboratory studies optimize materials around a single index, ignoring the mutual trade-offs brought by offshore high-temperature steam coupling environments [8,9].

1. Injectability is the fundamental precondition.

Offshore platforms lack large-scale dilution equipment, so a low-viscosity, uniformly dispersed initial fluid is set as the primary design threshold [17]. Increasing crosslinker dosage or particle loading improves plugging strength but sharply raises fluid viscosity, increasing the risk of pipeline plugging and prolonging construction cycles [13,20].

2. Deep migration targets inter-well steam channeling control.

Offshore dominant channels mostly develop in deep inter-well zones rather than near-wellbore regions [17]. Thermosensitive gels and modified expandable graphite realize delayed activation to extend migration distance, yet excessively low crosslink reactivity (for gels) or insufficient thermal expansion (for particles) reduces in situ retention efficiency, forming the universal migration-plugging trade-off bottleneck.

3. Balance between migration and in situ retention is the core design difficulty.

Long-distance migration is only a procedural means; stable retention and flow resistance establishment in target channels are the ultimate functional goals [14]. Excessively slow gelation leads to insufficient reservoir retention; rigid ultra-fine particles pass through pore throats without effective bridging. Parallel core flooding data of foam, gel and particle systems all prove that there is an optimal matching window between migration performance and retention capacity [14,24], yet quantitative matching criteria adapted to 200–350 °C offshore steam environments remain absent.

4. High-temperature anti-scouring stability is a unique rigid constraint.

Conventional static aging tests ignore cumulative thermo-mechanical fatigue under cyclic steam flushing. Ordinary partially hydrolyzed polyacrylamide (PAM) gels suffer rapid hydrolysis and network fracture under repeated shear; even phenolic resin gels with outstanding static heat resistance experience gradual structural integrity loss after multi-cycle scouring, leading to severe overestimation of service life by static evaluation methods [13,22].

5. Selective plugging and low formation damage guarantee long-term development value.

Profile control systems should selectively block high-permeability steam channels without polluting medium–low permeability oil-bearing layers [14]. Inorganic particles relying on physical bridging have excellent selective advantages, while excessive injection of bulk gels easily causes full-layer plugging and irreversible oil layer damage. Current responsive gel designs aim for targeted gelation in high-permeability zones, yet the permeability trigger threshold still requires further optimization [15].

6. Full-cycle engineering adaptability restricts formula popularization.

Offshore platforms have strict restrictions on raw material transportation, construction periods and operation safety [17]. Complex multi-component composite systems deliver balanced laboratory performance but complicate on-site mixing and increase construction costs, limiting large-scale offshore deployment. Single foam or simple polymer gel schemes have weaker comprehensive performance yet wider practical application due to convenient preparation.

In summary, unlimited optimization of any single performance indicator inevitably compromises other core demands. This mutually restrictive relationship lays the theoretical foundation for the multi-mechanism composite profile control systems discussed in Section 3.5.

## 3. Types and Mechanisms of Profile Control and Flooding Systems for Offshore High-Temperature Steam Flooding

### 3.1. Foam-Based Profile Control and Flooding Systems

As a typical mobility control medium for offshore steam flooding, thermal foam differs fundamentally from rigid plugging agents such as gels and inorganic particles in its action mechanism [10,11]. Rather than forming permanent flow barriers via crosslinked networks or particle bridging, foam relies on dense gas–liquid interfacial films to increase apparent flow resistance in high-permeability dominant channels, thus redistributing steam flow paths without completely blocking pore throats. This unique reversible regulation characteristic matches the demand for staged profile adjustment in the early–middle stage of offshore steam development, which has been validated by visualized micro-model tests and PVT viscosity measurement experiments [27,28].

#### 3.1.1. Mechanism of Foam-Based Systems

Two mainstream foam systems (N_2_ foam and CO_2_ foam) exert synergistic yet differentiated regulatory effects on steam propagation, a distinction rarely systematically compared in prior foam-focused reviews [19,29]. CO_2_ dissolves in crude oil to reduce interfacial tension and heavy oil viscosity simultaneously, while N_2_ forms a continuous heat-insulating layer in porous media to slow down formation heat loss and stabilize formation pressure. Multiple comparative PVT tests confirm that under the same gas mole fraction, CO_2_ delivers better viscosity reduction performance than nitrogen, but its interfacial film is more vulnerable to damage under high-salinity offshore formation water [19,28].

Notably, nearly all static laboratory evaluations overestimate the long-term performance of foam, as most existing single-factor experiments ignore continuous high-velocity steam shear and crude oil contamination [21,30]. Micro-model visualization data uniformly show that foam only maintains stable layered flow resistance under low-flow-rate static conditions; once subjected to cyclic steam scouring, gas–liquid films rupture rapidly, leading to sharp attenuation of flow regulation capacity. This static-dynamic performance deviation is an inherent defect of foam media, which has not been fully discussed in previous foam reviews [24,31].

**Figure 2:**
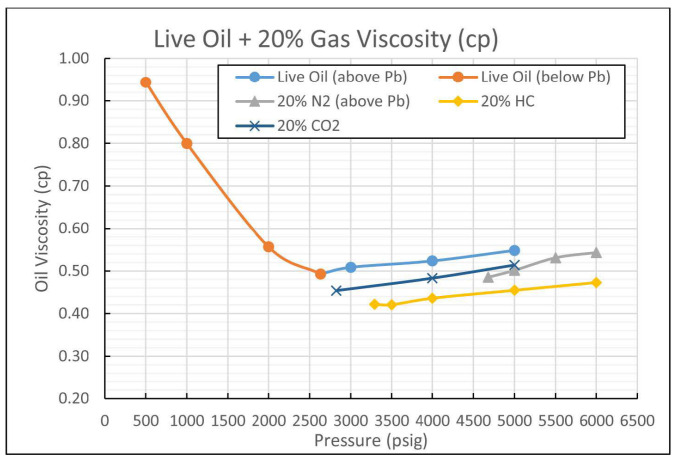
Viscosity reduction results of live oil mixed with 20% mole/mole different gases under variable pressure conditions. Reprinted from ref. [28] with permission from the publisher.

Figure 2 presents PVT comparative viscosity data of live oil mixed with different gases under variable pressure, reprinted from ref. [28]. The static test results reflect the instantaneous viscosity reduction effect of dissolved gas but cannot represent actual performance in offshore high-temperature steam flooding scenarios. In real reservoirs, continuous high-velocity steam scouring and high-salinity formation water accelerate foam film rupture and crude oil re-emulsification, so the actual duration of viscosity reduction is significantly shorter than static test results. This static-dynamic deviation is a common limitation of current foam performance evaluation.

The above static isothermal PVT test only reflects the instantaneous viscosity reduction effect of dissolved gas. In actual offshore high-temperature steam flooding scenarios, continuous high-velocity steam scouring and high-salinity formation water will accelerate foam film rupture and crude oil re-emulsification. As a result, the actual duration of the viscosity reduction effect is significantly shorter than the static test results, which constitutes a common static-dynamic evaluation deviation in current foam performance research [21,30].

#### 3.1.2. Advantages and Inherent Limitations of Foam Systems

Evaluated against the six-dimensional unified framework proposed in Section 2.2 (injectability, deep migration, thermal stability, anti-scouring, selective plugging, engineering economy), foam shows prominent differentiated advantages and irreconcilable defects compared with gel and particle media.

The core merit of foam lies in its excellent injectability and flexible staged regulation capacity [12,32]. Field pilot tests from multiple domestic heavy oil blocks show that nitrogen foam requires simple on-site gas–liquid mixing equipment, which is compatible with the narrow construction window of offshore platforms. It can achieve short-term steam diversion without permanent reservoir damage, and the oil-steam ratio can be increased by approximately 50% within a short construction cycle [12]. Unlike gels with the risk of near-well premature crosslinking, foam maintains full fluidity before entering high-permeability channels, realizing uniform deep migration without blockage risks.

However, cross-literature comparison of long-cycle steam flooding experiments reveals fatal drawbacks restricting its independent offshore application [18,30]. First, high temperature and offshore high salinity severely damage gas–liquid interfacial films; most conventional foams lose regulatory efficacy within 1–3 steam injection cycles, far shorter than the service life of phenolic resin and thermosensitive gels [2,21]. Second, crude oil components on reservoir surfaces accelerate foam coalescence and rupture, further shortening its effective action period, a factor seldom incorporated into early foam laboratory screening standards [30]. Third, foam only achieves temporary flow redistribution without forming persistent high-resistance structures; after gas injection stops, steam channeling rebounds rapidly, making foam incapable of treating mature inter-well dominant channels formed after long-term steam erosion [11,31].

Parallel dual-core flooding experiments further quantify the performance gap between foam and gel systems under identical high-temperature reservoir conditions [24]. Under a permeability ratio of 2:1 and a 300 °C steam environment, foam improves low-permeability core recovery by 25.20%, but this incremental effect fades quickly after multiple steam rounds; by contrast, gel systems maintain stable sweep expansion over long injection cycles. This contrast clearly explains why foam can only act as an auxiliary agent rather than a primary deep-plugging medium for offshore mature steam-channeling reservoirs.

#### 3.1.3. Offshore Applicability Boundary Analysis

Combined with reservoir development stages and offshore multi-coupling harsh conditions, the applicable scope of foam can be clearly defined via horizontal comparison with other profile control media. Foam is only suitable for the early and middle development periods of offshore steam flooding, where reservoir heterogeneity exists, yet continuous inter-well dominant steam channels have not fully formed [10,32]. At this stage, short-cycle foam injection realizes gentle steam profile adjustment to delay the formation of large-scale channeling, matching its characteristic of temporary mobility control.

For late-stage offshore reservoirs with mature, long-distance inter-well steam channels, single foam cannot provide persistent flow resistance and fails to restrain long-term steam breakthrough, so foam-gel composite modification becomes an inevitable optimization direction [9,33]. Existing composite foam research mainly uses polymer or thermosensitive gel to reinforce gas–liquid films and improve thermal and salt resistance; however, such composite schemes increase formulation complexity and offshore construction difficulty, weakening the original injectability advantage of foam [34].

Field-scale SAGD data further prove the limitation of single foam systems [10]. Although SAGD reservoirs achieve high overall recovery under ideal homogeneous conditions, once large-scale gravity override channeling forms, foam’s anti-scouring performance cannot meet the demand for long-term heat utilization improvement under offshore ultra-high-temperature continuous injection. Previous foam reviews mostly summarize onshore SAGD field effects without distinguishing offshore platform construction restrictions and higher formation salinity constraints, leading to over-optimistic judgment on foam’s offshore application potential.

#### 3.1.4. Summary

Foam systems occupy a unique position in the classification framework of offshore profile control media but have clear non-universally applicable boundaries. Their core value is low-cost, flexible staged mobility adjustment relying on reversible gas–liquid interfacial resistance, with unmatched injectability for short-term offshore construction. However, the inherent vulnerability of foam films under high temperature, salinity and cyclic steam shear leads to insufficient long-term stability, a fatal defect that single foam formulations cannot eliminate through simple formula optimization.

Compared with high-strength gel and selective particle plugging agents, foam cannot independently solve deep, mature inter-well steam channeling problems in the middle–late offshore development stage. The future research direction of foam media must focus on gel/nanoparticle composite film reinforcement to enhance dynamic anti-erosion performance, and foam can only be positioned as an auxiliary component of multi-mechanism composite profile control systems rather than a universal standalone plugging material for offshore high-temperature steam flooding reservoirs.

### 3.2. Gel-Based Profile Control and Flooding Systems

Gel systems form continuous 3D network structures via polymer-crosslinker reactions, delivering durable permeability reduction in dominant steam channels, and are the preferred medium for mature inter-well channeling treatment.

Different from petroleum engineering-oriented reviews that treat gels as simple plugging agents, this section subdivides high-temperature gel systems into six categories from the perspective of polymer material science: conventional PAM gels, phenolic resin crosslinked gels, nanocomposite reinforced gels, double-network (DN) hydrogels, thermosensitive smart gels, and degradable reversible gels. Their crosslinking mechanisms, thermal rheological properties and degradation laws under cyclic steam scouring are systematically compared, matching the material research scope of *Gels*.

#### 3.2.1. Conventional Polyacrylamide (PAM) Gels

Conventional PAM gels use partially hydrolyzed polyacrylamide (HPAM) as the main agent and metal ions (Cr^3+^, Al^3+^, Zr^4+^) as crosslinkers, forming loose single-layer networks via coordination bonds.

The crosslinking reaction follows a dynamic equilibrium process. The network densifies as temperature rises to 80–120 °C, with the steady-state storage modulus (G’) reaching 10–30 Pa·s and exhibiting typical elastic gel characteristics. Above 140 °C, severe hydrolysis of amide groups occurs, causing dissociation of coordination bonds and rapid network collapse; G’ drops by over 70% within 3 days, accompanied by obvious syneresis.

For offshore applications, PAM gels feature low raw material cost and good injectability (initial viscosity 20–60 mPa·s) but can only withstand temperatures ≤ 140 °C. Core flooding tests confirm their short-term plugging effect in porous media [35], but under 200–350 °C continuous steam scouring, the network structure degrades completely within one injection cycle. Cross-literature statistics show that the dynamic plugging retention rate is only 31–42% after 6 PV steam flushing, making it only applicable for auxiliary shallow plugging in low-temperature offshore blocks.

#### 3.2.2. Phenolic Resin Crosslinked Gels

Phenolic resin gels use low-molecular-weight phenolic prepolymers as crosslinkers, which react with amino groups on modified polyacrylamide chains to form rigid covalent crosslinking networks. This system is currently the most widely applied high-temperature gel variant in onshore thermal recovery projects [13,22].

At 180–260 °C, the storage modulus (G’) reaches 50–100 Pa·s and remains stable for 60–125 days due to the high bond energy of covalent bonds [36]. At 260–300 °C, G’ decreases slowly by 10–15% after 30 days of aging, and the network structure collapses completely above 300 °C.

Static high-temperature resistance is outstanding, with 93–98% plugging efficiency and 83–91% dynamic retention after 6 PV steam scouring at 260 °C. However, two main limitations restrict its offshore application: first, the high initial viscosity of phenolic prepolymers (125–260 mPa·s) raises the risk of near-wellbore accumulation, and fast crosslinking kinetics at high temperatures make deep migration difficult to control; second, conventional static oven aging tests generally overestimate field service life, as repeated thermal shock and high-speed fluid shear under cyclic steam injection cause cumulative fatigue damage to the gel network. Field practice shows that after 6 steam cycles, the actual residual plugging rate is approximately 40% lower than the static aging value [22].

#### 3.2.3. Nanocomposite Reinforced Gels

Nanocomposite gels introduce inorganic nanoparticles (nano-silica, nano-clay, graphene oxide) into the polymer crosslinking system as physical crosslinking nodes and reinforcing fillers, forming organic-inorganic hybrid networks combining chemical covalent bonds and physical hydrogen bonds.

Nanoparticles inhibit thermal movement of polymer chains and delay bond hydrolysis [37]. At 190–270 °C, G’ reaches 30–60 Pa·s, with only 10–15% mass loss after 30 days of aging at 260 °C. Reversible breakage of physical crosslinking points dissipates shear energy, giving better anti-fatigue performance than pure chemical gels.

They balance thermal stability and injectability, suitable for medium-temperature offshore blocks (180–220 °C). The main limitation is that nanoparticles are prone to agglomeration in high-salinity formation water (TDS > 30,000 mg/L), requiring surface modification to improve suspension stability.

#### 3.2.4. Double-Network (DN) Hydrogels

##### Material System Definition

DN hydrogels consist of two interpenetrating networks: a rigid high-crosslinking first network and a flexible low-crosslinking second network. The “sacrificial bond” mechanism (rigid bonds break preferentially to dissipate energy while the flexible network maintains integrity) endows the gel with both high strength and high toughness [38].

At 185–280 °C, G’ reaches 40–80 Pa·s, with fracture energy 10–100 times higher than single-network gels. The dynamic plugging retention rate is 80–89% after 6 PV steam scouring, as large elastic deformation avoids brittle fracture under high-speed shear.

The main offshore limitations are the complicated two-step preparation process and high raw material cost, which increase the difficulty of on-site deployment on platforms.

#### 3.2.5. Thermosensitive Intelligent Gels

Thermosensitive gels contain temperature-sensitive functional groups (e.g., PNIPAM) and undergo reversible sol–gel phase transitions when temperature exceeds the lower critical solution temperature (LCST), driven by hydrophobic interactions between molecular chains.

Below LCST, the solution has low viscosity (10–35 mPa·s) and excellent injectability; above LCST, viscosity increases sharply by 3–4 orders of magnitude, with G’ rising to 20–50 Pa·s. The LCST can be tuned by adjusting monomer ratio to match reservoir temperature.

This system perfectly fits the offshore design concept of “deep migration first, in situ high-temperature activation”. However, its LCST has a narrow adjustable range and is highly sensitive to salinity and pH; high-salinity offshore formation water easily destroys the hydration layer and causes premature gelation [39]. Gel strength formed by physical crosslinking is also lower than covalent crosslinking systems.

#### 3.2.6. Degradable Reversible Gels

Degradable reversible gels introduce hydrolyzable/oxidizable bonds (ester, disulfide, acetal bonds) into the crosslinking network, which can degrade into small molecules under specific reservoir conditions to realize reversible plugging.

Before degradation, G’ reaches 20–40 Pa·s with 85–90% plugging efficiency; after complete degradation, fluid viscosity returns to below 10 mPa·s, and reservoir permeability recovers to over 90% of the original value. The degradation rate can be controlled from days to months by adjusting bond type and density [40].

They avoid permanent reservoir damage and are suitable for temporary profile adjustment in the early-middle stage. But thermal stability is poor, and the degradation rate is difficult to accurately control under complex offshore reservoir conditions.

The structural and performance differences among the six high-temperature gel systems are systematically compared in Table 2.

#### 3.2.7. Summary

No single gel system can simultaneously satisfy all performance requirements of offshore high-temperature steam flooding. Conventional PAM gels trade thermal stability for injectability; phenolic resin gels sacrifice migration capacity for high-temperature resistance; emerging nanocomposite, DN, and thermosensitive gels alleviate partial bottlenecks but still face engineering matching problems such as high-salinity stability and temperature window adaptation. Future development should focus on multi-functional crosslinking network design and composite modification to achieve balanced performance.

### 3.3. Particle-Based Profile Control and Flooding Systems

Different from foam’s reversible flow regulation and gel’s chemical crosslink plugging, particle systems construct flow resistance in high-permeability channels via physical migration, inter-particle bridging and in situ deposition. Dominated by inorganic solid materials, they possess intrinsic ultra-high thermal stability unmatched by organic polymer systems, making them uniquely adaptable to 200–350 °C cyclic steam environments offshore. This section divides particle materials into conventional rigid inorganic particles and thermally expandable graphite (EG) functional particles and compares their plugging mechanisms, performance characteristics and application boundaries under the six-dimensional evaluation framework.

#### 3.3.1. Core Plugging Mechanisms

Particle media rely on physical stacking rather than chemical crosslinking, forming selective blocking structures only in oversized pore throats of dominant channels without damaging low-permeability oil layers. Two typical types show differentiated action paths:

1. Conventional inorganic particles (fly ash, quartz powder, mineral microspheres).

They maintain stable solid morphology at constant high temperatures, migrate along high-permeability zones carried by steam, and form dense bridging deposits at pore throats. Parallel core tests prove that such materials achieve over 99% plugging efficiency in high-permeability cores but only 10% permeability reduction in low-permeability formations, showing excellent selective plugging capacity [14]. However, rigid particles lack deformability; mismatched particle size will either cause near-wellbore accumulation or pass through channels without forming effective resistance.

2. Thermally expandable graphite (EG) particles.

**Figure 3:**
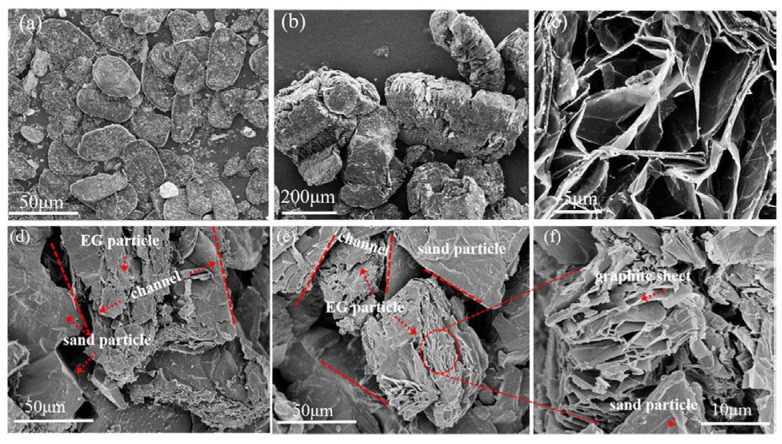
(**a**–**f**) Microscopic morphology of expandable graphite (EG) particles before and after thermal expansion. Reprinted from ref. [20] with permission from the publisher.

As a novel in-depth steam channeling control material, original EG particles have small size and good fluidity for long-distance inter-well migration in offshore large well spacing. When exposed to continuous high-temperature steam, the interlayer structure expands to form worm-like flexible aggregates, which stack tightly in dominant channels to build long-term anti-scouring barriers [20]. The coupled “deep migration + in situ expansion” mechanism effectively relieves the particle size matching bottleneck of traditional rigid particles. The microscopic morphology of EG particles before and after thermal activation is shown in Figure 3.

The optimal expansion morphology comes from static oven heating. In actual long-distance migration offshore, continuous steam shear will partially damage the interlayer structure in advance, resulting in lower actual expansion ratio and plugging strength than static test values, which is a common reason for the gap between laboratory effect and field performance.

#### 3.3.2. Performance Advantages and Inherent Contradictions

Evaluated by the six offshore screening standards, the two particle types have distinct advantages and unavoidable defects:

1. Conventional inorganic particles: Ultra-high temperature resistance up to 350 °C without thermal degradation; low raw material cost; outstanding selective plugging with minimal damage to medium–low permeability layers. The fatal defect is the strict particle-pore matching threshold: oversized particles deposit near wellbores and block injection pipelines, while ultra-fine particles cannot form effective bridging and lead to rapid steam channeling rebound.
2. EG particles: Dual advantages of small initial size for injection and post-expansion plugging capacity; stronger anti-scouring performance than rigid inorganic particles after thermal activation. The main limitations are the narrow expansion temperature window and high raw material cost, which restrict large-scale offshore batch construction.

A universal core contradiction exists in all particle systems: injectability and plugging strength cannot be optimized simultaneously. Smaller particle size ensures smooth injection and deep migration but sacrifices bridging capacity; larger size improves plugging efficiency but increases near-well deposition risk.

#### 3.3.3. Applicable Scenario Boundary Distinction

From the perspective of reservoir development stages, particle media have clear targeted application ranges:

1. Applicable scenarios: Middle–late steam flooding reservoirs with mature, large-scale deep inter-well dominant channels. Wide pore throats formed by long-term steam erosion cannot be fully blocked by foam or conventional polymer gels, while inorganic and EG particles maintain stable physical plugging under continuous high-temperature flushing, making them the preferred deep treatment medium for severe offshore channeling.
2. Inapplicable scenarios: Early reservoirs without obvious dominant channels. Small reservoir pore size easily causes near-well particle accumulation and formation damage, where foam’s gentle staged regulation is more suitable.
3. Cross-system comparison: Particles have permanent physical plugging capacity without failure after injection stops, but gel can form uniform networks in tiny fractures that particles cannot enter; foam has simpler construction and lower cost for short-term adjustment.

#### 3.3.4. Research Bottlenecks and Development Directions

##### Main Bottlenecks

1. Lack of quantitative particle size matching criteria targeting offshore 200–350 °C steam reservoirs; most existing models are derived from onshore low-temperature water flooding cores.
2. Insufficient dynamic evaluation: Most tests adopt static suspension observation, with few multi-cycle steam flooding models to characterize particle migration and loss law under long-term shear.
3. Scattered modification research: Separate studies improve suspension or expansion performance, lacking integrated modified particle systems balancing injectability, deep migration and long-term anti-scouring performance.

##### Future Optimization Directions

1. Graded compound particle design: Mix micro-fine and moderate-size inorganic powder to realize graded bridging and relieve single-size matching limitations.
2. Surface-modified EG: Introduce a hydrophilic/lipophilic coating to adjust the expansion trigger temperature, widen the applicable temperature range and reduce cost.
3. Particle-gel composite synergistic systems: Combine the deep migration advantage of particles and uniform crosslinking performance of low-concentration gels, which is a mainstream composite research trend (detailed in Section 3.5).

#### 3.3.5. Summary

Particle-based systems have incomparable ultra-high temperature resistance and selective layer protection advantages under severe offshore steam coupling conditions, especially suitable for deep dominant channel treatment in middle–late steam flooding blocks. However, all particle materials face the inherent contradiction between particle size, injectability and plugging efficiency. Follow-up research on graded compound particles, surface-modified thermally expandable materials and particle-gel composites is expected to balance migration and plugging performance and expand offshore application scope.

### 3.4. Thermally Responsive Profile Control and Flooding Systems

Different from foam’s temporary flow regulation and the passive plugging mode of gels/particles, thermally responsive materials take reservoir high temperature as an activation trigger rather than an environmental constraint [26,41], fundamentally resolving the universal contradiction of “near-well premature plugging vs. insufficient deep retention” in conventional single-component media. This section divides systems into three subtypes, compares their phase transition behaviors and comprehensive performance under the six-dimensional evaluation framework, and clarifies their differentiated application boundaries for offshore scenarios.

#### 3.4.1. Action Mechanisms

Three mainstream subtypes show distinct response paths under offshore steam temperature gradients:

1. Thermal solidification agents: Linear macromolecules maintain a low-viscosity fluid state at surface injection temperature (40–60 °C); after migrating to deep zones ≥180 °C, intramolecular aggregation and intermolecular entanglement form dense blocking layers without additional crosslinkers [41]. Activation depends only on reservoir heat, avoiding near-well premature crosslinking from mixing errors.
2. Single-chain thermosensitive gels: Contain temperature-sensitive hydrophobic side chains. Below the critical phase transition temperature (CPT), hydrated groups ensure high fluidity; above CPT, broken hydration layers trigger rapid molecular aggregation into elastic gel networks. The main limitation is the narrow adjustable CPT range.
3. Dual-network thermally responsive microgels: Composed of interpenetrating soft temperature-sensitive chains and rigid heat-resistant skeletons. Thermal stimulation triggers secondary crosslinking after deep migration; the rigid skeleton guarantees anti-scouring performance, while the soft chain flexibly controls the trigger threshold [26]. This structure balances migration capacity and plugging strength and is the most promising emerging branch aligned with *Gels’* scope.

**Figure 4:**
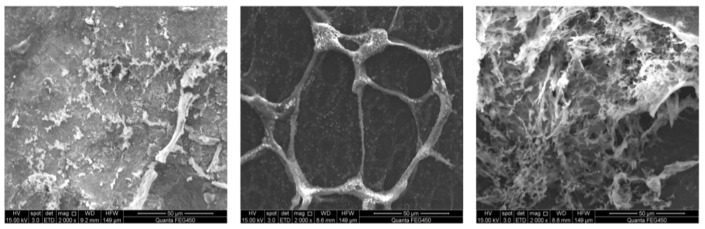
Scanning electron microscopy (SEM) images of conventional organic gel and composite thermoresponsive gel after long-term high-temperature aging. Reprinted from ref. [15], ACS Omega, under the terms of the CC BY 4.0 license.

Most existing mechanism tests adopt constant-temperature static ovens, failing to simulate the gradual temperature rise [2,15]. The microstructural morphologies of the conventional gel and the composite thermoresponsive gel after thermal aging are compared in Figure 4.

These static post-aging characterizations cannot reflect dynamic network evolution under cyclic steam shear. Under combined thermal hydrolysis and mechanical scouring, gel network pores will gradually expand and the skeleton will fragment, which cannot be fully presented by static post-test observation.

#### 3.4.2. Performance Comparison and Inherent Limitations

Evaluated by the six offshore screening standards, three subtypes show distinct trade-offs [15,16,26,41]:

1. Thermal solidification agents: Ultra-low initial viscosity, excellent injectability, simple deployment without extra crosslinkers, wide activation range (160–320 °C). Limitation: formed blocking layers lack elastic networks and peel off easily under multi-cycle steam impact with shorter service life.
2. Single-chain thermosensitive gels: Low cost, mature synthesis route, finely adjustable CPT via monomer ratio. Limitation: narrow temperature adaptation window; ±15 °C deviation from design CPT will cause premature aggregation or ineffective plugging; high salinity destroys hydrated side chains in advance.
3. Dual-network microgels: Optimal overall balance, flexible trigger range, and outstanding anti-scouring performance with >98% plugging efficiency after 8 PV steam flushing [16]. Limitations: complex two-step synthesis, high raw material cost, strict requirements for injection water ion composition.

All thermally responsive materials achieve the “deep migration first, in situ activation” breakthrough, but no subtype can simultaneously satisfy all offshore conditions. Current modification mostly optimizes single indicators, lacking a multi-index balanced matching system for high-salinity, multi-cycle steam environments.

#### 3.4.3. Cross-System Applicability Boundary Analysis

Thermally responsive systems have clear scenario positioning differentiated from other media:

1. Suitable: Early-middle stage reservoirs with large well spacing, uniform medium permeability, and no mature wide steam channels. They maintain full fluidity during long-distance migration and form targeted resistance only in deep high-temperature zones, perfectly matching preventive deep profile control demand.
2. Unsuitable: Late-stage reservoirs with large-scale wide inter-well channels. Their activated network thickness is limited compared with high-dose phenolic gels and EG particles, unable to completely block oversized pore throats formed by long-term erosion.
3. Cross-medium comparison: They have permanent plugging capacity unlike foam, avoid near-well premature gelation unlike conventional gels, and form flexible networks compatible with tiny pore throats unlike rigid particles.

#### 3.4.4. Research Bottlenecks and Future Directions

##### Core Bottlenecks

1. Insufficient Temperature–salinity coupling research. Most formulas are optimized under single-temperature fresh water conditions, lacking systematic tests for offshore high-salinity and temperature gradient coexistence [17,41].
2. Imperfect dynamic evaluation system. Static aging data dominate performance judgment, with few multi-cycle steam simulations to characterize network failure under long-term shear.
3. No quantitative field matching model between reservoir temperature field, well spacing and material trigger concentration, relying on empirical screening [26].

##### Development Trends

1. Salt-resistant molecular modification to widen the applicable salinity window.
2. Optimized dual-network structures to balance trigger flexibility and anti-scouring performance and reduce costs for large-scale offshore application.
3. Standardized dynamic evaluation criteria based on multi-cycle core flooding to narrow lab-field performance deviation.
4. Thermally responsive-particle composite systems combining deep migration advantage and ultra-high temperature stability for middle–late stage reservoirs with moderate channel scale.

#### 3.4.5. Summary

Thermally responsive systems fundamentally solve the near-well premature plugging defect of conventional agents by using reservoir heat as an activation trigger and are the preferred preventive deep regulation material for early-middle offshore steam flooding blocks. Dual-network microgels achieve the optimal overall performance, most consistent with the *Gels* journal orientation. Current limitations include narrow salinity adaptation, over-reliance on static evaluation and high cost. Future research should focus on salt-resistant modification, dynamic evaluation system construction and multi-mechanism composite design.

### 3.5. Composite Profile Control and Flooding Systems

The core motivation for composite systems lies in the inherent performance trade-offs of single foam, gel, particle and thermally responsive media under offshore high-temperature, high-salinity and cyclic steam coupling environments [9,34]. Composite systems break through the performance ceiling of single components via multi-mechanism synergy, integrating respective strengths while offsetting inherent defects, and have become a mainstream research direction for offshore thermal recovery. Existing schemes are divided into two technical routes: gel-reinforced foam composites and organic-inorganic hybrid composites.

#### 3.5.1. Synergistic Mechanisms

The core motivation for composite systems lies in the inherent performance trade-offs of single foam, gel, particle and thermally responsive media under offshore high-temperature, high-salinity and cyclic steam coupling environments [9,34]. All single materials face irreconcilable limitations: foam lacks long-term anti-scouring stability; conventional gels suffer from premature near-wellbore gelation; rigid particles are restricted by strict pore size matching; single thermosensitive materials have narrow temperature adaptation windows. Through multi-mechanism synergy, composite systems break through the performance ceiling of single components, integrating respective strengths while offsetting inherent defects, and have become a mainstream research direction for offshore thermal recovery EOR materials.

1. Gel-reinforced foam composite system.

This system adopts segmented injection or one-pot compounding of surfactant foam and low-concentration polymer gel. Foam provides excellent injectability and early steam mobility regulation, while crosslinked gel molecules adsorb on gas–liquid film surfaces to thicken interfacial layers and inhibit high-temperature oil-induced coalescence [33,34]. Comparative tests confirm that gel networks extend the effective service cycle of foam from 1 to 2 steam rounds to more than 5 cycles, fundamentally alleviating the short-life defect of single foam under high-salinity conditions. However, excessive gel dosage will increase working fluid viscosity and weaken deep migration performance.

2. Organic-inorganic hybrid composite system.

This category includes particle-gel and thermosensitive-nanocomposite subtypes. Inorganic particles (expandable graphite, nano-clays) act as physical reinforcing nodes embedded in organic crosslinked networks, enhancing gel skeleton rigidity and shear resistance under continuous steam flushing [15,20]; thermally responsive microgels compounded with inorganic fillers can adjust the thermal trigger window while retaining long-distance migration advantages. Unlike foam-based composites, such materials focus on mitigating thermal hydrolysis failure of pure organic polymers at 200–350 °C, yet the suspension stability of inorganic solids in high-salinity formation water remains a restrictive factor.

Notably, most current formula optimizations are conducted under fixed salinity and simulated oil laboratory conditions. For offshore formation water with high divalent cation content, high-concentration salt ions will compress the surfactant electric double layer and weaken the polymer thickening effect, resulting in partial failure of the composite film structure. Single-factor laboratory optimization results therefore cannot be directly applied to complex offshore formation environments.

#### 3.5.2. Performance Comparison and Inherent Limitations

Evaluated by the six unified screening standards, three mainstream composite schemes show complementary advantages and new bottlenecks brought by component superposition [9,33,34]:

1. Gel-reinforced foam composite: Retains foam’s low-viscosity injection advantage for simple offshore construction; significantly improves high-temperature and oil resistance of gas–liquid films, extending the effective regulation period. Limitations: narrow formulation matching window; still unable to form high-strength plugging barriers for mature large-scale inter-well channels.
2. Particle-gel organic-inorganic hybrid: Inorganic particles enhance gel network thermal stability and reduce thermal decomposition loss; gel solves the near-well deposition defect of single rigid particles, realizing coordinated deep migration and in situ bridging plugging. Plugging efficiency remains above 95% after more than 8 PV steam scourings. Limitations: complicated preparation procedures; nano/inorganic particles are prone to agglomeration in high-salinity water, causing pipeline fouling risks.
3. Nano-modified thermally responsive composite gel: Inorganic nano-fillers widen the temperature trigger range, adapting to offshore reservoirs with uneven vertical temperature distribution; it retains low-viscosity deep migration characteristics before thermal activation, and the hybrid network strengthens post-gelling anti-erosion performance. Limitations: The high nano raw material cost and complex surface modification process limit mass field deployment.

Composite systems can effectively offset single performance shortcomings of individual media, but component superposition will inevitably generate new mutually restrictive contradictions. No universal composite formula can satisfy all offshore reservoir temperature, salinity and construction conditions simultaneously.

#### 3.5.3. Applicable Scenario Boundaries

Combined with reservoir development stages and offshore construction conditions, the application scope of composite systems is clearly divided:

1. Gel-reinforced foam composite: Suitable for early-middle stage blocks with mild heterogeneity, undeveloped large stream channels, and high requirements for simple construction. Not recommended for late-stage reservoirs with long-distance dominant channels.
2. Particle-gel hybrid composite: Suitable for middle–late stage blocks with mature deep steam channels, 220–350 °C reservoir temperature and medium-high formation salinity. It is the preferred composite scheme for severe channeling treatment.
3. Nano-thermally responsive composite gel: Suitable for newly commissioned offshore well groups with large well spacing, medium reservoir temperature and complex vertical temperature gradient, focusing on preventive deep profile adjustment.

Cross-system comparison shows that composite systems are not complete substitutes for single media, but targeted upgrades for specific scenarios that balance conflicting performance indicators.

#### 3.5.4. Research Bottlenecks and Development Trends

##### Main Bottlenecks

1. Most matching experiments adopt single salinity and fixed temperature laboratory environments, lacking multi-factor coupling simulation of actual offshore formation conditions (variable temperature + high TDS + crude oil contamination).
2. Insufficient dynamic evaluation research; performance judgment mostly relies on static aging observation, with few multi-cycle steam flooding models to track particle settlement, gel interfacial detachment and film rupture attenuation laws under long-term shear.
3. Engineering adaptability research lags behind laboratory formula optimization; raw material cost, mixing process complexity and platform equipment matching are rarely evaluated, ignoring offshore high-cost, short-operation-window constraints.

##### Future Optimization Directions

1. Multi-factor coupled formula screening: carry out orthogonal experiments of temperature–salinity–oil contamination ternary coupling to widen the applicable matching window.
2. Dynamic standardized evaluation system construction: take multi-cycle cyclic steam core flooding as the core evaluation method to reduce lab-field performance deviation.
3. Low-cost simplified composite design: optimize inorganic filler dosage and polymer concentration, reduce compound preparation steps, and develop economical composite schemes adapted to large-scale offshore batch injection.
4. Targeted composite customization: design differentiated foam-gel/particle-thermosensitive composite formulations according to reservoir development stages to realize classified precise material matching.

Table 3 systematically and quantitatively compares all mainstream profile control systems under unified offshore high-temperature simulation conditions. All data are normalized to eliminate experimental parameter inconsistencies across different references, providing an intuitive horizontal comparison of core performance indicators and offshore engineering applicability.

#### 3.5.5. Summary

Composite systems make up for the inherent performance defects of single media via multi-mechanism synergy, breaking through the single-index trade-off bottleneck that individual materials cannot solve. Nevertheless, component compounding introduces new restrictive contradictions such as a narrow formulation window, a complex preparation process and a high raw material cost. Future research should focus on multi-factor coupled formula optimization, dynamic steam evaluation standard establishment, low-cost simplified design, and formulating differentiated composite schemes for different offshore reservoir development stages.

### 3.6. Dynamic Laboratory Evaluation Methods and Future Research Directions

The significant discrepancy between static plugging efficiency and dynamic retention rate in Table 3 confirms that conventional static evaluation systems cannot accurately reflect the actual service performance of profile control agents under long-term cyclic steam scouring in offshore reservoirs. Static high-temperature aging tests only simulate constant-temperature thermal degradation, ignoring the combined effects of high-velocity steam shear, cyclic thermal loading, high-salinity formation water and crude oil contamination, generally overestimating the effective service life of materials by 2–3 times [20,27]. Establishing standardized dynamic evaluation methods adapted to offshore multi-coupling harsh conditions is critical to narrow the lab-field performance gap.

#### 3.6.1. Limitations of Conventional Static Evaluation Methods

Current performance screening mostly relies on static oven aging tests and single-cycle core flooding experiments, with three inherent defects for offshore steam flooding scenarios:

1. Static aging cannot simulate progressive structural degradation under dynamic steam shear. For organic gels, long-term high-velocity scouring induces both thermal hydrolysis and mechanical shear fracture of crosslinked networks, leading to faster strength attenuation than static aging [22]; for inorganic particles, continuous fluid scouring destroys stacked bridging structures and causes gradual particle loss, which cannot be reflected by static suspension tests [20].
2. Single-factor constant-condition evaluation ignores the multi-coupling effect of complex offshore reservoirs. Actual offshore formations feature coexisting high temperature, high salinity, crude oil contamination and temperature gradient, while most laboratory tests optimize formulas under single temperature and fresh water conditions, leading to sharp performance decline in field application [15,26].
3. Lack of standardized test protocols for multi-cycle steam injection. Offshore heavy oil development requires dozens of steam injection cycles, while current evaluation mostly adopts single injection-displacement mode, which cannot characterize cumulative fatigue damage after repeated thermal shock and shear scouring. Studies have verified that after 6 steam cycles, the plugging strength of conventional phenolic gels decreases by more than 40% compared with single-cycle results [13], which is highly consistent with the dynamic retention data in Table 3.

#### 3.6.2. Standardized Dynamic Evaluation Protocols for Offshore Conditions

Targeting the above defects, a three-module standardized dynamic evaluation system should be established to cover the whole process of material injection, deep migration, in situ activation and long-term scouring:

1. Multi-cycle cyclic steam core flooding test. Taking six pore volume (PV) cyclic steam scouring as the standard benchmark, test dynamic plugging retention rate, anti-scouring pressure-bearing capacity and long-term permeability change. The test should simulate actual offshore injection-production rhythm, including alternating steam injection and shut-in soaking stages, to characterize cumulative fatigue damage under repeated thermal loading [20,21]. This module can directly quantify static-dynamic performance deviation, replacing traditional static plugging efficiency as the core evaluation index [42].
2. Temperature–salinity–oil coupling aging test. On the basis of conventional high-temperature aging, simultaneously introduce high-salinity formation water and crude oil components to simulate comprehensive degradation effects of complex offshore formations. Set gradient salinity (20,000–40,000 mg/L) and gradient temperature (180–350 °C) to test material adaptation windows, avoiding mismatch between laboratory-optimized formulas and actual formation conditions [15,26].
3. Gradient temperature deep migration simulation test. Use long cores with temperature gradient distribution to simulate the gradual temperature rise process from wellbore to deep inter-well zones and characterize migration distance, activation timing and plugging position of thermally responsive materials and expandable particles. This module is specially designed for offshore large well spacing scenarios, which can effectively verify premature gelation/expansion near the wellbore and make up for the evaluation blind spot of deep migration performance in conventional short core tests [20,26].

#### 3.6.3. Key Future Research Priorities

Combined with current technical bottlenecks and offshore engineering demands, future research should focus on four core directions:

1. Molecular modification of salt-resistant thermally responsive composite gels. Introduce anti-ion interference functional groups and construct organic-inorganic hybrid networks to widen the salinity adaptation window and improve high-temperature shear resistance. The dual-network thermally responsive microgel system is the most promising technical route, but its preparation cost and field-matching degree still need further optimization [15,26].
2. Low-cost multi-mechanism composite formula design. On the premise of ensuring balanced performance, simplify the compounding process and reduce raw material costs to meet the requirements of large-scale batch injection on offshore platforms. The particle-gel hybrid system with a simple mixing process is the preferred scheme for popularization at this stage [20,34].
3. Unified dynamic evaluation standards and field matching prediction models. Take multi-cycle cyclic steam core flooding as a mandatory evaluation procedure, unify test parameters and indicators across laboratories, and establish a quantitative prediction model between reservoir parameters, construction parameters and treatment effect. A long-term reservoir numerical simulation model considering thermal-shear coupling degradation can be further constructed to realize quantitative prediction of profile control effect over multiple cycles.
4. Differentiated material screening criteria based on development stages. Formulate targeted selection guidelines corresponding to early prevention, mid-stage adjustment and late deep channeling treatment to realize precise matching between materials and reservoir conditions and avoid blind plugging agent selection in field construction.

## 4. Conclusions

This paper constructs a six-dimensional unified evaluation system guided by the “flow control–diversion–enhanced sweep” theory to systematically compare foam, gel, particle, thermally responsive and composite profile control media for offshore high-temperature steam flooding. Cross-literature critical comparison reveals three irreconcilable core constraints restricting field application: reservoir heterogeneity-induced deep steam channeling, the universal trade-off between deep migration and plugging strength of all materials, and unique high-cost, narrow-window offshore engineering limitations. In addition, prevailing static laboratory tests consistently overestimate material long-term performance under cyclic dynamic steam shear, forming a widespread research gap in existing studies.

Horizontal comparison clarifies clear stage-specific application boundaries for each medium: foam is only fit for early-stage temporary mobility adjustment; conventional polyacrylamide gels degrade rapidly under ultrahigh temperature, while phenolic, nanocomposite and double-network gels, together with thermally responsive variants, represent high-performance gel routes aligned with the scope of *Gels*; inorganic particles excel at high-temperature selective plugging yet suffer strict pore-matching limits; composite systems offset single-material drawbacks but bring complicated deployment and narrow formulation windows. No universal agent can satisfy all offshore working conditions simultaneously.

Three prominent research deficiencies are summarized: over-reliance on static evaluation, lack of multi-index balanced material design, and disconnection between molecular material research and offshore engineering demands. Correspondingly, future work should focus on four targeted directions: developing salt-resistant thermally responsive composite gels, optimizing low-cost multi-mechanism compound formulas, establishing standardized dynamic steam flooding evaluation protocols, and building reservoir-stage differentiated material screening criteria.

Overall, single-component profile media have inherent defects under offshore coupled harsh environments. Subsequent material development should prioritize balancing injectability, deep migration and thermal stability, with standardized dynamic testing systems as essential support to lift steam sweep efficiency and heavy oil recovery in offshore thermal recovery projects.

## Figures and Tables

**Table 1:** Comparison between this review and previously published relevant review articles.

Review Category & Representative References	Main Research Object	Core Research Content	Existing Obvious Limitations	Unique Advantages of this Review
High-temperature gel material reviews [8,9]	Land thermal recovery polymers and phenolic gel materials	Static thermal stability, crosslinking agent optimization, onshore field plugging performance	1. Ignore dynamic steam shear and cyclic thermal aging failure;	1. Systematically classify 6 high-temperature gel systems from a polymer science perspective and compare their crosslinking mechanisms and thermorheological properties under uniform thermal stress for the first time;
			2. No separate classification of nanocomposite, double-network and thermosensitive gels;	2. Focus on material degradation under continuous dynamic steam flow;
			3. No discussion on offshore platform construction restrictions	3. Fully integrate the matching analysis of offshore engineering conditions
Steam foam review papers [10,11]	N_2_/CO_2_ foam for auxiliary steam flooding	Foam generation mechanism, static stability, single foam field application	1. Foam, gel and particle systems are discussed separately without horizontal comparison;	1. Establish the first unified quantitative comparison database covering 5 categories and 11 types of profile control systems under offshore 200–350 °C steam conditions;
			2. Lack unified quantitative performance evaluation indexes;	2. Clarify that foam is only used as an auxiliary flow control agent in the early and middle stages of offshore steam flooding
			3. Not suitable for ultra-high-temperature continuous steam scouring in offshore reservoirs	
General thermal EOR conformance control reviews [6,12]	All chemical plugging agents for onshore heavy oil reservoirs	Brief introduction of basic mechanisms of gel, particle and foam	1. No distinction between onshore and offshore harsh working conditions;	1. Take the unique multi-coupling harsh environment of offshore reservoirs as the core research background;
			2. Lack of in-depth analysis of gel network structure from a material perspective;	2. Add in-depth gel material chemistry analysis matching the scope of *Gels*;
			3. No independent discussion on dynamic laboratory evaluation methods	3. Set up an independent special subsection for dynamic steam performance evaluation
Onshore heavy oil profile control reviews [13,14]	Water shutoff and profile control technology for onshore cyclic steam stimulation	Onshore reservoir adaptability, single-well construction technology	1. Ignore the demand for long-distance deep migration under large well spacing in offshore oilfields;	1. Take “deep migration first, in situ high-temperature activation” as the core design logic for offshore systems;
			2. Insufficient research on continuous high-temperature steam erosion resistance;	2. Quantitatively compare the long-term anti-scouring performance of all types of profile control systems
			3. No systematic sorting of thermally responsive delayed deep activation systems	
Mini-reviews on single thermally responsive materials [15,16]	Thermosensitive gel for conventional high-temperature reservoirs	Phase transition mechanism of single thermogel	1. No horizontal comparison with foam and inorganic particle systems;	1. Unify five single systems and composite multi-phase systems into one complete analysis framework;
			2. Lack of a summary of multi-component composite synergistic systems;	2. Propose a composite system optimization route targeting severe deep steam channeling in offshore reservoirs
			3. Not combined with the actual engineering practice of offshore steam flooding	

Table Notes: (1) Representative reviews/studies for each category are selected from the reference list of this paper. (2) The limitations of each category are summarized based on the research scope and core content of the cited references [6,8,9,10,11,12,13,14,15,16].

**Table 2:** Material structure and performance comparison of six types of high-temperature gel systems. Test reference condition: gelation curing at corresponding applicable temperature, long-term aging at 260 °C, TDS = 30,000 mg/L.

Gel System Category	Dominant Crosslinking Bond Type	Max Applicable Temperature (°C)	Steady-State Storage Modulus G’ (Pa·s)	Dominant Thermal Degradation Mechanism	Core Material Advantages	Core Inherent Limitations
Conventional PAM Gel [9,22]	Metal ion coordination bond	≤140	10–30	Amide group hydrolysis + coordination bond dissociation	Low raw material cost, simple preparation, good initial injectability	Severe thermal hydrolysis above 140 °C: network collapses rapidly under steam scouring
Phenolic Resin Crosslinked Gel [13,22,36]	Covalent methylene bridge bond	200–300	50–100	Methylene bridge fracture + polymer chain thermal oxidation	Outstanding static high-temperature resistance, high plugging strength	High initial viscosity, poor deep migration capacity and fast crosslinking at high temperature
Nanocomposite Reinforced Gel [37]	Chemical covalent bond + physical hydrogen bond	190–270	30–60	Organic chain hydrolysis + nanoparticle agglomeration	A hybrid skeleton delays thermal degradation and improves anti-fatigue performance	Nanoparticles agglomerate easily under high salinity; strict dispersion control required
Double-Network (DN) Hydrogel [26,38]	Interpenetrating dual covalent networks	185–280	40–80	Progressive fracture of rigid first network	High strength and high toughness, resistant to steam shear brittle fracture	The two-step preparation process is complicated and has a high raw material cost
Thermosensitive Intelligent Gel [15,39]	Hydrophobic interaction (physical crosslinking)	175–290	20–50	Disintegration of hydrophobic aggregates + loss of small molecular chains	Ultra-low viscosity before activation, perfect for deep migration	High salinity sensitivity, low gel strength compared with covalent crosslinking systems
Degradable Reversible Gel [8,40]	Hydrolyzable covalent bond (ester/disulfide bond)	160–240	20–40	Degradable bond breakage + overall network depolymerization	Reversible plugging, minimal permanent reservoir damage	Degradation rate is difficult to control; poor long-term thermal stability

Table Notes: (1) Steady-state storage modulus (G’) is the stable value after complete gelation at the optimal applicable temperature of each system, reflecting the elastic strength of the gel network. (2) Thermal degradation mechanisms and performance parameters are summarized based on long-term high-temperature aging and cyclic steam scouring experiments in cited references. (3) Data sources for each gel system: conventional PAM gel [9,22]; phenolic resin crosslinked gel [13,22,36]; nanocomposite reinforced gel [37]; double-network hydrogel [26,38]; thermosensitive intelligent gel [15,39]; degradable reversible gel [8,40]. (4) This table focuses on material-level structural and rheological comparison; the engineering application performance of each gel system under unified offshore conditions is detailed in Table 3.

**Table 3:** Quantitative performance comparison of offshore high-temperature steam flooding profile control systems.

System Category	Temp Range (°C)	Max TDS (mg L^−1^)	Static Plugging (%)	Dynamic Retention (6 PV, %)	Incremental Oil Recovery (%)	Initial Viscosity (mPa·s)	Adaptability Score	Core Pros/Cons
3.1 Foam Systems [10,21,23,24,28]								
N_2_/CO_2_ Foam	170–240	15,000–20,000	66–76	23–36	6–12	2–7	3	Excellent injectability
								Film ruptures under high temp/salinity
3.2 Non-Thermo-Responsive Gels [9,13,22,36,37,38]								
Conventional PAM Gel	≤140	15,000	91–95	31–42	8–15	22–62	2	Low cost
								Severe hydrolysis above 140 °C
Phenolic Resin Gel	200–300	35,000	93–98	83–91	12–20	125–260	4	Outstanding static heat resistance
								Poor deep migration
Nanocomposite/DN Non-Responsive Gel	185–280	30,000–32,000	92–97	76–89	15–22	65–140	3.6–3.8	Reinforced thermal skeleton
								Nanoparticle agglomeration
3.3 Particle Systems [14,20]								
Rigid Inorganic Mineral Particles	200–350	42,000	96–99	88–95	10–18	6–18	4.2	Ultrahigh temperature resistance
								Strict particle−pore matching
Expandable Graphite (EG) Particles	200–350	40,000	97–99	91–96	18–25	5–16	5	Deep migration before expansion
								High raw material cost
3.4 Thermally Responsive Systems [15,26,41]								
Thermal Solidification Agent	160–320	28,000	88–93	68–78	12–18	4–12	4	Ultra-low injection viscosity
								Peeling under multi-cycle steam
Single-Chain/DN Thermo-Responsive Gel	175–320	22,000–32,000	90–96	72–89	15–22	11–35	3.7–4.5	Balanced migration & plugging
								Narrow salinity window
3.5 Composite Multi-Mechanism Systems [20,33,34,42]								
Gel-Reinforced Foam Composite	180–250	24,000	72–82	48–60	12–18	8–22	3.5	Extended foam service life
								Narrow formulation window
Particle-Gel Organic-Inorganic Hybrid	210–330	38,000	95–99	87–95	20–28	32–72	4.3	Synergistic heat resistance
								Multistep mixing procedure
Nano-Modified Thermo-Responsive Composite	195–325	34,000	93–97	81–90	18–26	14–38	4.4	Wide trigger temperature range
								High nanomaterial cost

Table Notes: (1) All data are averaged interval values extracted from parallel core-flooding and long-term thermal aging experiments in cited references. (2) Dynamic retention rate reflects long-term anti-scouring performance; incremental oil recovery is obtained from parallel core flooding tests under a permeability ratio of 2:1. (3) Offshore adaptability score (1–5) integrates temperature–salinity tolerance, construction complexity, raw material cost and field stability. (4) Core reference sources: Foam systems [10,21,23,24,28]; non-thermo-responsive gel systems [9,13,22,36,37,38]; particle systems [14,20]; thermally responsive systems [15,26,41]; composite systems [20,33,34,42].

## Data Availability

No new data were created or analyzed in this study. Data sharing is not applicable to this article.
