# Correction: Liu et al. Action Mechanism, Research Progress and Development Trend of High-Temperature Steam Flooding and Profile Control/Flooding Systems. *Gels* 2026, *12*, 586

**DOI:** 10.3390/gels12100880

**Published:** 2026-09-30

**Authors:** Yigang Liu, Jianhua Bai, Xiaodong Han, Qiuxia Wang, Hongwen Zhang, Hongyu Wang, Jinxiang Liu, Yifei Gao, Xianpei Yin

**Affiliations:** 1 CNOOC Key Laboratory of Offshore Heavy Oil Thermal Recovery, Tianjin 300452, China; 2 Key Laboratory of Enhanced Oil and Gas Recovery of Ministry of Education, Northeast Petroleum University, Daqing 163318, China

In the original published article [1], Zilong Liu was mistakenly included in the author list. According to the four ICMJE authorship criteria, Zilong Liu only completed trivial literature sorting and reference formatting work, and made no substantial contributions to the conception, design, analysis, writing or final approval of this review paper. He was mistakenly added during manuscript submission. With full written consent from Zilong Liu and all other nine co-authors, we remove Zilong Liu from the author list. The corrected author list is shown above.

The author contributions have been revised accordingly.

Conceptualization, J.L. and Y.L.; methodology, Y.G. and X.Y.; validation, J.B., X.H. and Q.W.; formal analysis, Y.G.; investigation, Y.L., H.Z. and H.W.; resources, J.B., X.H. and Q.W.; data curation, Y.G. and X.Y.; writing—original draft preparation, Y.L. and Y.G.; writing—review and editing, J.L., J.B. and H.Z.; visualization, Y.G. and X.Y.; supervision, J.L.; project administration, J.L.; funding acquisition, J.B. All authors have read and agreed to the published version of the manuscript.

The scientific conclusions, data, analysis and all research content of this review remain unaffected by this correction. This correction was approved by the Academic Editor. The original publication has also been updated.
